# Acknowledgement to Reviewers of *Pharmaceutics* in 2013

**DOI:** 10.3390/pharmaceutics6010078

**Published:** 2014-02-28

**Authors:** Pharmaceutics Editorial Office

**Affiliations:** MDPI AG, Klybeckstrasse 64, CH-4057 Basel, Switzerland

The editors of *Pharmaceutics* would like to express their sincere gratitude to the following reviewers for assessing manuscripts in 2013:

Armenante, Piero M.
Asbill, C. Scott
Bayes-Garcia, Laura
Cao, Sheng
Carlson, Harold E.
Cheung, Chi Wai
Childs, Scott L.
Cho, Cheon-Weon
Christensen, Dennis
Cui, Zhengrong
Dash, Alekha
Di Stefano, Antonio
Espinoza, Luis R.
Ewert, Kai K.
Fahnestock, Margaret
Fang, Jia-You
Felton, Linda A
Francia, Giulio
Gallardo, Carmen R
Ghafourian, Taravat
Gräber, Peter
Grégoire, Sébastien
Grice, Jeffrey E.
Halberstadt, Adam L.
Halila, Sami
Heath, Timothy
Horiuchi, Shin
Jain, Rahul
Jayaraman, Muthusamy
Kan, Chi-Wai
Kelly, Julie
Kim, Sanghoon
Lee, Beom-jin
Li, Mingzhong
Li, Xiaojuan
Liu, Xin-ming
Lockridge, Oksana
Loguercio, Carmela
Lu, Wei Yue
Luo, Juntao
Maas, Mario
Maggio, Edward T.
Mannervik, Bengt
Martini, Silvia
Moos, Philip J.
Morimoto, Yuji
Moriyama, Yoshinori
Mozafari, Marcel R.
Muller, Michael
Nakayama, Tsutomu
Norén, G. Niklas
O'halloran, Thomas V.
Ohya, Yuichi
Ouyang, Defang
Panczyk, Tomasz
Panisello, Cinta
Papineni, Arathi
Patterson, Adam V.
Pepeu, Giancarlo
Peura, Lauri
Picard, Martin
Pillay, Viness
Priemel, Petra
Rades, Thomas
Rautio, Jarkko
Rigas, Basil
Ristori, Sandra
Rosado, Catarina
Shen, Yaochun
Shim, Chang-Koo
Sikorski, Alexander
Singh, Mogie
Sinico, Chiara
Skoda, Erin
Song, Xingfu
Tajber, Lidia
Tamanoi, Fuyuhiko
Tileli, Vaso
Torres, Vukosava Milic
Vita, Roberto
Waldmeier, Felix
Watson, Douglas S.
Wei, Kuo-chen
Were, Lilian, M.
Wilson, Bill
Winter, Jarrold C.
Xue, Min
Yang, Eddy S.

